# Vector control programs in Saint Johns County, Florida and Guayas, Ecuador: successes and barriers to integrated vector management

**DOI:** 10.1186/1471-2458-14-674

**Published:** 2014-07-02

**Authors:** Diana P Naranjo, Whitney A Qualls, Hugo Jurado, Juan C Perez, Rui-De Xue, Eduardo Gomez, John C Beier

**Affiliations:** 1 Department of Public Health Sciences, University of Miami Miller School of Medicine, Miami, FL 33136, USA; 2 Servicio Nacional de Control de Enfermedades Transmitidas por Vectores Atrópodos, Ministerio de Salud Pública, Guayaquil, Ecuador; 3 Instituto Nacional de Salud Pública e Investigación, Ministerio de Salud Pública del Ecuador, Guayaquil, Ecuador; 4 Anastasia Mosquito Control District, St. Augustine, FL 32080, USA; 5 Facultad de Ciencias Médicas, Universidad Católica Santiago de Guayaquil, Guayaquil, Ecuador; 6 University of Miami, Clinical Research Building #1027, 1120 NW 14th ST, Miami, FL 33136, USA

**Keywords:** Mosquito control, Vector-borne diseases, Decision analysis, Health policy, SWOT analysis, Surveillance systems

## Abstract

**Background:**

Vector-borne diseases (VBDs) and mosquito control programs (MCPs) diverge in settings and countries, and lead control specialists need to be aware of the most effective control strategies. Integrated Vector Management (IVM) strategies, once implemented in MCPs, aim to reduce cost and optimize protection of the populations against VBDs. This study presents a strengths, weaknesses, opportunities, and threats (SWOT) analysis to compare IVM strategies used by MCPs in Saint Johns County, Florida and Guayas, Ecuador. This research evaluates MCPs strategies to improve vector control activities.

**Methods:**

Methods included descriptive findings of the MCP operations. Information was obtained from vector control specialists, directors, and residents through field trips, surveys, and questionnaires. Evaluations of the strategies and assets of the control programs where obtained through SWOT analysis and within an IVM approach.

**Results:**

Organizationally, the Floridian MCP is a tax-based District able to make decisions independently from county government officials, with the oversight of an elected board of commissioners. The Guayas program is directed by the country government and assessed by non-governmental organizations like the World health Organization. Operationally, the Floridian MCP conducts entomological surveillance and the Ecuadorian MCP focuses on epidemiological monitoring of human disease cases. Strengths of both MCPs were their community participation and educational programs. Weaknesses for both MCPs included limitations in budgets and technical capabilities. Opportunities, for both MCPs, are additional funding and partnerships with private, non-governmental, and governmental organizations. Threats experienced by both MCPs included political constraints and changes in the social and ecological environment that affect mosquito densities and control efforts. IVM pillars for policy making were used to compare the information among the programs. Differences included how the Ecuadorian MCP relies heavily on the community for vector control while the American MCP relies on technologies and research.

**Conclusion:**

IVM based recommendations direct health policy leaders toward improving surveillance systems both entomologically and epidemiologically, improving community risk perceptions by integrating components of community participation, maximizing resources though the use of applied research, and protecting the environment by selecting low-risk pesticides. Outcomes of the research revealed that inter-sectorial and multidisciplinary interventions are critical to improve public health.

## Background

Mosquito control programs (MCPs) have been developed and operate to protect individuals from vector-borne diseases (VBDs) [1]. As mosquito populations and pathogens are ecologically diverse and ever changing [2,3], it is critical to evaluate the performance of MCPs within a comprehensive framework covering multiple environments and threats. Because VBDs and MCPs diverge in varying settings and countries, control specialists must be aware of collective guidelines for policy making [4,5].

To address these challenges, the World Health Organization (WHO) has proposed a universal concept of Integrated Vector Management (IVM), which once applied to MCPs, aims to be a “rational decision-making process for the optimal use of resources in the management of vector populations, so as to reduce or interrupt transmission of vector-borne diseases” [6,7]. An applied IVM structure can maximize the use of resources by making vector control more efficient, cost effective, and ecologically sound [8]. It is noteworthy that such IVM methods are aimed to significantly reduce vector populations even more so when applied to strengthen capacity within sectors in developing countries. In Uganda, agricultural and health sector collaborations to control both pests and vectors proved to foster knowledge sharing among sectors, thus offering complimentary expertise of rural service delivery to malaria susceptible agricultural communities [9]. However, to demonstrate how IVM strategies work in different settings, vector control specialists and scientists need to report on what is occurring in their respective region with accompanying epidemiological and entomological data [10,11].

Aside from adequately reporting disease cases, control professionals and vulnerable communities may also lack the power to convince decision-makers of the public health importance of scaling up vector control [10]. Commitment from national authorities implies investment of resources and time in creating effective policies and communication networks with control professionals, education specialists, health care leaders, and the community. Given the that agreements need to be reach by multiple parties for decision making, vector control actions or policies can be delayed [12,13]. In other instances, country leaders may fail to mobilize communities for preventive action regarding potential threats because the community may not perceive that they are at risk [14]. A way to address these issues and present them to decision makers is by measuring the factors that influence the MCP effectiveness, including interactions of the environment with the sociopolitical system and the extent of protection of the public health of the population. An effective and simple tool used for decision making for business is the SWOT analysis, which identifies the strengths, weaknesses, opportunities, and threats of an organization. The tool helps label internal and external factors that affect the performance of business programs in order to help avoid failures as well as improve efficiency of every day operations [15]. Understanding the key factors that drives successful health systems, like MCPs, is key to measure the effectiveness of the organization.

In order to demonstrate that implementation of IVM can improve vector control at a country level, this paper provides comparative information on the organizational and operational structure, and current control strategies functioning in the Anastasia Mosquito Control District (AMCD) in Saint Johns County Florida, U.S. and the *Servicio Nacional de Control de Enfermedades Transmitidas por Vectores Atrópodos* (SNEM) in Guayas, Ecuador. The aims of this research are 1) to deliver an SWOT of these MCPs based on IVM guidelines and 2) to document lessons learned from the programs of AMCD and SNEM providing beneficial information for MCPs and policymakers, and 3) suggest ways to apply IVM to other international settings.

## Methods

### Study sites

#### Anastasia mosquito control district of saint Johns County

AMCD is located at the northeastern side of Florida in Saint Johns County and controls over 43 nuisance and vector species of mosquitoes [16]. Like many Floridian cities, the climate is subtropical with a warm-dry and a hot-wet season. AMCD generates revenue for services though taxes to the community, and an elected board of commissioners regulates decisions. The District currently serves 210,000 residents with an annual budget of 2.7 million dollars allocated for staff and operational supplies [17,18].

#### Servicio Nacional de control de enfermedades transmitidas por vectores atrópodos

SNEM is located in the seaport city of Guayaquil, Guayas, on the southwest coast of Ecuador. The climate is tropical with two seasons that vary from humid-hot to dry-warm. The institution is a subgroup of the Ministry of Health and the institution’s decision-making is centralized. SNEM was designed to stimulate economic development and was established with the help of international funding in 1949 through the United States Agency for International Development (USAID) and the Inter-American Development Bank. The initial aim was to protect the health of banana farmers facing malaria epidemics [19].

The province of Guayas is divided into 16 urban and 5 rural neighborhoods [20] and vector control operations of SNEM are deployed to locations where human cases are reported [21]. SNEM operates on an annual budget of $3 million primarily allocated for multiple VBDs and serves over 2.5 million people [22].

### Field trips and surveys, SWOT analysis, and the integrated vector management pillars

#### Field trips and interviews and surveys

We accompanied technicians on daily routine operational field visits to residential properties for house inspection and larviciding. During these visits, we retrieved information through personal communications with the staff, field workers, and community members. Ancillary data, such as public records, were used to document the organizational structure, human and operational resources, and entomological and epidemiological profiles.

Surveys were conducted with the directors and employees of the institutions to identify the internal and external parameters affecting the MCPs. In our study, we used a similar methodological framework as detailed in the research of Impoinvil and colleagues [4].

#### SWOT analysis

This study used the SWOT approach to identify the different competences of both MCPs. We evaluated the structure of control operations, staff, assets, law formulation, capacities, financial resources, and overall efficacy to identify strengths and weaknesses of the programs.

The internal factors were ordered as strengths and weaknesses and such factors were associated to the presence or absence of resources that belong to the organization. For instance, efficient data management systems are strengths while, shortage of personnel and equipment are weaknesses. Internal factors can improve or hinder the operations of the MCP. In contrast, external factors are not dependent on the organization itself. These factors are classified as opportunities and threats, and such consist of the surrounding political, social, climate, and community environments that affect the decisions of the MCP [15]. For instance, city infrastructure and regulatory bodies influence the MCPs in different forms. Assertive political will favoring vector control could create opportunities to facilitate the delivery of educational campaigns, while poor sewage services could become a threat affecting control activities by multiplying mosquito larval habitats.

#### Integrated vector management pillars for policy making

The IVM questionnaires and recommendations for policy-making by Van den Berg et al. were included as part of our methods [23]. We focused on addressing the IVM elements that structure successful vector control programs: advocacy and social mobilization, collaborative efforts, integrated approaches, evidence-based decision making, and capacity building. We highlighted the most important findings in each category and provided a recommendation suitable for the setting in which the MCP functions.

The Board of Commissioners of AMCD provided verbal consent to conduct this study and facilitated administrative public information of the District. Additional verbal agreement was obtained from the Director of the SNEM. Human data and human subjects were not used for this research. However, ethical considerations for the use of non-human data were discussed. The chief authorities of AMCD and SNEM approved the materials and methods used in this manuscript.

## Results

### Operational and organizational structure

#### Anastasia mosquito control district

The daily routine of the operational staff includes surveillance of mosquito populations, environment (rainfall, temperature, tide) and presence of West Nile virus (WNV), eastern equine encephalitis virus and St. Louis encephalitis virus through laboratory confirmed seropositive conversion of blood samples from sentinel chickens. The decision to treat an area is based on the evidence that the virus is circulating in an area, thereby field personnel enforces treatments where the virus is reported. Treatments are also based on threshold densities of adult mosquito populations.

Daily, the MCP staff performs source reduction, larviciding, and adulticiding to keep mosquito populations at levels that do not threat the quality of life of the residents. Lower mosquito bites per minute and reducing breeding sites may also diminish the vector and nuisance mosquitoes. Maps and records of areas that have been treated are kept and later reported to the Florida Department of Agriculture and Consumer Services (FDACs), which regulates pesticide applications. Mosquito population data has been tracked since the establishment of the District in 1948.

Staff training is required to maintain their Florida Public Health Pesticide Applicators license. The District runs training programs and workshops throughout the year that provides employees with the continuing education credits needed to maintain their license. Further, technicians acquire knowledge on new trends for mosquito control, calibration procedures, peak mosquito activity, and ground and air applications [24].

**Figure 1:**
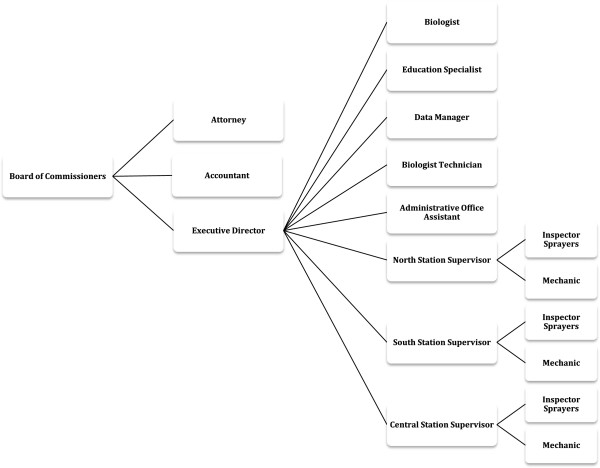
**Organizational diagram of the Anastasia Mosquito Control District**[25]**.**

The organizational structure of the District is presented in Figure 1[25]. The Executive Director is the lead decision maker in regards to operational control as much as administration. Operationally, the Director relies on both a biologist and the field supervisor, to execute the control activities efficiently. A community elected board that promotes the interests of the residents regulates decision-making. Administrative staff keeps track of all operations and makes sure the entity complies with regulations through record keeping.

#### Servicio Nacional de control de enfermedades transmitidas por vectores stropodos

Operationally, SNEM relies on epidemiological surveillance of malaria and dengue cases, where reports are accessible through the technological data platform, *Sistema Integrado de Vigilancia Epidemiológica* (SIVE *alerta*). Surveillance information reported from health centers to the Ministry of Health is made public to inform vector control specialists, as much as citizens, on the number of cases by province. Complementary to surveillance, SNEM protects residents from vectors through the use of community monitoring of VBD, specifically strengthening local vector control once a case is reported in a household.

The Guayas based program oversees the activity of multiple infectious disease systems and related vector control. Main efforts by SNEM target *Aedes aegypti* mass source reduction activities due to the ongoing reports of dengue fever. Furthermore, the Guayas-based institution monitors other parasitic diseases through the control of species of *Triatoma* (Hemiptera: Reduviidae) vectors of *T. cruzi* infections (i.e. Chagas disease), in addition to species of *Lutzomyia* (Diptera: Psychodidae) as vectors of *Leishmania* parasites, which appears as sporadic cases as Cutaneous Leishmaniasis in this region.

Furthermore, the Guayas-based institution monitors other parasitic diseases through the control of species of *Triatoma* (Hemiptera: Reduviidae) vectors of *T. cruzi* infections (Chagas disease), in addition to species of *Lutzomyia* (Diptera: Psychodidae) as vectors of *Leishmania* parasites which appears as sporadic cases of Cutaneous Leishmaniasis in rural areas of the region.

SNEM inspectors complete door-to-door larviciding to educate the community on source reduction. A *visitador*, a technician or an inspector sprayer, is a community member trained in a central station who takes charge of educating other community leaders in suburban areas of the province. Personnel also organize field collections of mosquito larvae for entomological identification and insecticide resistance testing purposes. Education specialists are part of the mosquito control network and are trained to support the home assessments. Government health staff performs both microscopic diagnoses of malaria pathogens (*Plasmodium vivax and P. falciparum*). Dengue diagnostics is made at public or private health centers. Clinicians order ELISA for detection of antigen NS1 if the patient has less than 5 days of dengue related symptoms; if symptoms last more than 5 days, detection of IgM antibodies is done [26].

**Figure 2:**
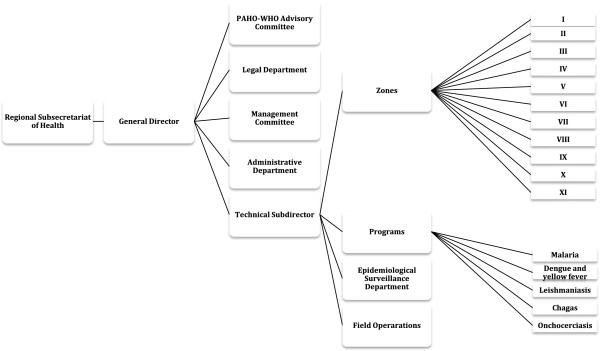
**Organizational diagram of the *Servicio Nacional de Control de Enfermedades Transmitidas por Vectores Atrópodos*** [27]**.**

Organizationally, the Technical Subdirector makes the main judgments in regards to field operations in Guayas and countrywide; however two different authorities assigned by the central government of Ecuador regulate the aforementioned position. These authorities are the regional Subsecretary of Health and the General Director. Additionally, WHO-Pan American Health Organization (PAHO) committee suggests control and policy approaches to vector abatement operations. The Technical Subdirector supervises six different administrators who operate the different branches to control different types of VBDs in the region. Figure 2 presents detailed information on the organization of SNEM [27].

### SWOT analysis

SWOT analysis revealed critical issues with decision-making, financing, and delivery of the different control strategies. Common issues of the programs are laid out as 1) Strengths: Research based operations and community participation, 2) Weaknesses: Epidemiological-entomological surveillance and insecticide resistance, 3) Opportunities: Capacity building, research collaborations and improved municipal services, 4) Threats: Political, geographical situation, environmental and social constraints.

#### Strengths: Research based operations and community participation

One of the identified strengths of AMCD is their research-based operational program, which aims to conduct tests on equipment and pesticides ensuring the optimal use of resources; i.e. minimum amounts of active ingredients, best solvents for pesticides, etc. If funding is not sufficient, the District can generate additional money from grants and direct funds towards the AMCD applied research program. Concurrently, trials and information regarding insecticide use and applications are analyzed and published making the findings of the District available to the public. Record keeping of the entomological data has allowed the District to make evidence based decisions to better manage pesticide treatments, thus ensuring AMCD has justification to treat.

Moreover, monitoring of key entomological and environmental conditions such as the presence of circulating WNV, fluctuations in mosquito populations, and changes in weather patterns, are considered additional strengths because AMCD can plan ahead when vector-human contact increases. The infrastructure based on entomological monitoring supports AMCD to make accurate assessments of virus activity, mosquito population densities, and environmental surveillance.

Internal assets include the educational and training components, which includes the training and re-training of entomologists, educators, field workers, and administrative staff. This ensures the correct use of resources, pesticides, and proper knowledge of mosquito habitat development by personnel. Inside training influences the scientific community from junior scientists to experienced researchers who may access the facility to conduct experiments in a variety of ecosystems.

Other important features include up to date technologies to increase community feedback and improve targeted treatments. An implemented service request system allows direct calls, emails, and use of social media from the residents to alert AMCD staff that mosquitoes are becoming a nuisance in a specific area. Additionally, GPS technologies in the spray trucks track the routes, time, and place of chemical applications facilitating the production of reports to the regulating body, FDACs.

SNEM uses a large network of *visitadores* who facilitate tracking of human cases and population-based approaches while conducting routine home visits. Population-based control allows broad surveillance, targeted treatments, and dissemination of educational material in rural areas and hard to reach neighborhoods. Numerous laboratories and trained technicians working in facilities all around the cities multiply the outreach capacity of the organization. SNEM concentrates their efforts in rapid case detection through malaria microscope diagnosis, and further tests household members who where in contact with the person(s) infected. Weekly follow-up on the residents allows the execution of outbreaks contingency plans, especially when dealing with malaria cases.

An added benefit to SNEM is their interaction with international and national organizations as well as effective communication at a citizen level. SNEM leaders participate with non-governmental organizations (NGOs) by taking part in global projects such as the Amazon Malaria Initiative funded by the USAID, IVM strategy workshops for specialists sponsored by WHO and PAHO, and the Roll Back Malaria program; previous mentioned programs make training of professionals more affordable and accessible [28]. At the national level, SNEM and political leaders (e.g., ministries of health and mayors) codirect *mingas* in neighborhoods for source reduction purposes. *Minga* is an organized activity through which residents are called out publicly to perform an action on a designated area that will benefit their own community. During peak dengue season, the Mayor of the city of Guayaquil broadcasts a call for a *minga* in a particular neighborhood and those residents come out at an established time to perform source reduction activities, such as emptying water containers or cleaning the streets [29].

#### Weaknesses: Epidemiological-entomological surveillance and insecticide resistance

The lack of an epidemiological surveillance system represents one of the main weaknesses at AMCD. In the case of VBDs, the lag time between case diagnosis at a health center and reporting by the Florida Department of Health to the District may be too long or not even reported. Data systems are absent in regards to reporting human mosquito borne disease cases to the MCP, thus citizens and local physicians may not be educated to screen local viruses in patients reporting fever, which may lead to underreporting of cases of WNV and dengue fever. Table 1 illustrates the different pathogens both institutions survey.

**Table 1:** Entomological and epidemiological profile of most important pathogens and mosquito species controlled at Anastasia Mosquito Control District and Service of National Malaria Eradication

**Study site**	**Disease ( *Pathogen *)**	**Vector**	**Reservoir *host (dead-end host)***	**Ecology**	**Epidemiology**
AMCD	Dengue (*Flavivirus)*	*Aedes aegypti, Ae. albopictus*	Human (*human*)	Suburban and urban	Epidemic
	West Nile virus (*Flavievirus*)	*Culex nigripalpus, Cx. quinquefasciatus*	Birds (*human-horses*)	Suburban	Endemic
	Eastern equine encephalitis (*Alphavirus*)	*Culiseta melanura*	Birds (*human-horses*)	Suburban	Endemic
SNEM	Dengue (*Flavivirus*)	*Aedes aegypti*	Human (*human*)	Domestic suburban and urban	Endemic, epidemic
	Malaria (*Plasmodium spp.*)	*Anopheles albimanus, An. pseudopunctipennis, An. darlingi, An. punctimacula*	Human (*human*)	Rural and suburban settings	Endemic
	Yellow fever (*Flavivirus*)	*Aedes aegypti*	Human (*human*)	Rural in Amazon region	Endemic

Moreover, high insecticide costs and limited number of active ingredients registered for use may represent a major setback for the program since rigorous control interventions with the same active ingredient can create resistance due to high selective pressures on the targeted mosquito population [30]. Efforts to reduce pathogen-carrying mosquitoes have been based on the use of insecticides and such efforts are ineffective in the presence of insecticide resistance within *Anopheles spp.* or *Aedes spp.* populations [31,32]. Unforeseen environmental changes can worsen the above-mentioned situation [33].

Budget limitations at AMCD prevent hiring more field or administrative staff thereby hindering the ability of the District to respond to service requests within 24 hours. The District currently answers requests within 48–72 hours. Shortness of managerial staff can also delay required administrative or bureaucratic demands made by the Board of Commissioners, state laws, environmental agencies, and the FDACS which are mandatory for field operations.

In contrast to the AMCD, the absence of entomologists, entomological training or entomological surveillance is one of the main weaknesses at SNEM. The leaders of the Guayas-based program are usually medical doctors that may not have sufficient epidemiological training to apply public health approaches, strategic planning for entomological surveillance and response to environmental threats is absent as well. Insufficient entomological training for *visitadores* is a limitation as they may not be aware of potential breeding sites or correct mosquito identification. Even though SNEM hires a large workforce, a number of *visitadores* are trained just once and may not be re-trained or hired for full time positions, thereby reducing the efficiency of control strategies.

SNEM also lacks a number of active ingredients for larviciding purposes. This restricts the ability of SNEM to counteract pesticide-resistance. Pesticide resistance testing is not done regularly and when it is done in the laboratory setting, field trials for these pesticides are not conducted to corroborate the lab work.

Other practical measures such as aerial spraying and adulticiding are absent in the Guayas based program. Available spraying trucks are limited, making the application of adulticides the last resort of their control strategy. Larviciding without other supporting forms of control may result in a city less resilient to outbreaks, due to the lack of supplies and equipment.

#### Opportunities: Capacity building, research collaborations and improved municipal services

The educational program at AMCD informs their constituents on personal protection knowledge and promotes community awareness of VBD risk through the participation of the District in community fairs and school visits. These interactions between residents and MCPs are also seen at the scientific level. Research exchanges between scientists at the United States Department of Agriculture, universities, and international institutions increase the expertise and knowledge of AMCD professionals. Additional collaboration with government entities and universities is done through annual AMCD-hosted workshops.

The Ecuadorian government has capitalized from its oil industry and has made investments in roads, infrastructure, municipal services, healthcare, and capacity building. Regarding healthcare, increased investments provides better access to care, treatment, and patient follow ups, therefore reducing human reservoirs of the pathogens. In regards to capacity building, the MCP of Guayas has the opportunity to collaborate with the Ecuadorian *Instituto Nacional de Salud Pública e Investigación* (INSPI) and the initiative, *Prometeo*, which has the possibility to enhance SNEM programs through integrating research-based operations. *Prometeo* belongs to the Ecuadorian Secretaría Nacional de Educación Superior, Ciencia, Tecnología e Innovación (SENESCYT), and has received funding representing the equivalent of 1.86% of the country’s Gross Domestic Product; the most an American country has invested in education. Through SENESCYT, country leaders aim to educate young individuals in doctorate programs and post-graduate fellowships and promote capacity building and technology transfer [34].

At best, opportunities are found through multiple sources of funding for training and education. In regards to training, international entomological workshops help SNEM personnel to learn about current trends for vector control. Such training takes place in countries abroad and is funded by non-governmental agencies. The government also allocates funding for local training of technicians and community-based approaches, including home visits. Furthermore, the Ecuadorian government invests in educational campaigns on dengue prevention, which are broadcasted via newspapers, radio, television, and Internet advertisements.

#### Threats: Political, geographical situation, environmental and social constraints

Threats to AMCD fall within political, administrative, and environmental demands. Politically, turnover of the elected officials or board members may delay communications between the District and regulating bodies and delay control actions. Increments in the amount of the bureaucracy, administrative workload, and record keeping are administrative threats. External constrains placed on the District to meet state pesticide reporting compliance escalates the amount of work required for the program to function.

Urban, ecological, and geographical threats are also present due to city growth and increasing service request demands. These requests may not be answered promptly because, despite city expansion, the District is not expanding and personnel are still limited. Slow responses in mosquito control treatments may result in the dissatisfaction of residents. Another factor includes the geographical location of Saint Johns County, which is surrounded by both salt and fresh water, resulting in diverse mosquito populations.

Climate events also affect the SNEM and they represent the main threats to the program. Climate phenomenon such as El Niño Southern Oscillation (ENSO) can influence VBD transmission by increasing raining events and flooding, multiplying breeding sites as well as providing mosquito populations with optimal temperature and humidity levels to survive longer periods of time [35]. In 2012, Zambrano et al. demonstrated that ENSO was the climate factor responsible for an 150% increase in the incidence of dengue in Honduras [36]. Guayas, due to its tropical climate and geographical location faces cyclical epidemics of dengue. The number of dengue cases in the city has increased in the past decade and the presence of such climate phenomenon is expected every five to seven years.

Another challenge for SNEM is the limited ability to treat dengue hot spot areas due to security reasons. The rapid expansion of the city in the last 10 years is causing increased inner migration from rural to urban areas of Guayaquil, which is the largest area covered by SNEM. Overcrowding leads to the development of slums or *invasiones* that are usually lack of municipal services and can become a hub of vectors and diseases. Crime rates in the aforementioned marginal neighborhoods are higher; thereby spray trucks drivers or larviciding teams may not feel safe to perform their jobs. Likewise, some residents, due to security fears, may not allow *visitadores* to enter their properties.

### Integrated vector management and driving forces for policy-making: resources and decision-making

Generally, limited resources are the number one driving force for decision-making on both MCPs. High cost of insecticides is the major source of spending for both programs combined with lackluster efforts to improve insecticide resistance problems and VBD reduction, especially in the Guayas setting. SNEM relies heavily in larviciding alone while adulticiding methods are rarely used. Seasons with high mosquito densities can result in an increase in MCPs spending on both insecticide and overtime personnel requirements. AMCD depletes budget and resources in the summer time for which they allocate moneys for strengthening aerial and land treatments as much as manpower.

**Figure 3:**
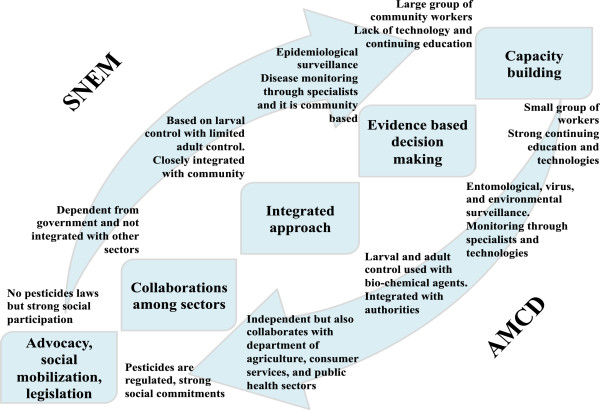
Findings of the SWOT analysis at Anastasia Mosquito Control District (AMCD) and the Service of National Malaria Eradication and Control of Arthropods (SNEM) in regards to Integrated Vector Management elements.

The IVM five elements approach is seen n different degrees in AMCD and SNEM. Our main findings are described in Figure 3 and such address the main questions of the previously discussed IVM recommendations of Henk van den Berg et al. [23].

## Discussion

### Recommendations

#### Evidence based decision-making

The quick response of AMCD to environmental threats is based upon the knowledge of their entomologists, employees, and leaders. An entomological component is needed in systems like the SNEM to aid in decision-making processes. Furthermore, implementation of such entomological components may not require outstanding investments. One inexpensive option is to use oviposition traps made of water filled plastic cups to collect eggs laid by mosquitoes for surveillance of *Aedes* vectors [37]. In Northern Italy, an entomological surveillance method implemented during Chikungunya outbreaks drove a massive employment of oviposition traps for monitoring of *Aedes* mosquitoes in 227 municipalities. The strategy strengthened their response system because of their improved entomological surveillance for disease prevention, furthermore egg density indices allow better evaluation of infestation rates at lower operational costs in *Ae. aegypti* population surveillance [38].

Supplementary to entomological approaches, epidemiological data systems are also essential. Health sectors may support data management systems to complement entomological information, thereby creating a clearer picture of VBD risk [39]. For instance, making site-specific records of human cases and matching these records with historical data can enhance decisions for control of vectors and associated pathogens. It can also produce geo-referenced information on the time and place of infections [40]. SIVE *alerta* system in Guayas provides the MCP with the time and location of human cases so they can activate vector control efforts accordingly. For all the programs, surveillance systems need to be backed by data monitoring technologies that are readily available to field workers and public health leaders.

Another factor affecting MCPs includes budgetary issues especially when outbreaks occur, stressing the importance of making cost-effective investments to manage epidemics. One of these approaches could be increasing funding towards preparedness initiatives and early warning systems as ways to forecast the epidemics. Epidemics management is expensive and depletes funds of health care systems much more than investing on vector control. Research done in Cuba demonstrates that the total economic health expenditure per resident doubles during dengue outbreaks season compared with seasons of no transmission. The high costs are attributed to hospitalizations and case management but not on enforced vector population control [41]. If policy makers focus in vector control surveillance, outbreaks could be predicted earlier and overall disease burden, both financially and with respect to the individual, can be lessened.

#### Integrated approaches

To ensure the rational use of available resources we need to establish a multi-disease control approach [10]. This approach may entail collaboration across a broad spectrum of control programs allowing the identification of common factors that have not been controlled for maximum benefit [42]. Extrapolating malaria control success to dengue control may be difficult, however programs like SNEM can try to increase communication among their different branches for vector control trying to perform not only malaria diagnosis on site, but dengue clinical diagnosis or referrals as well.

Multiple strategy integration may also effectively diminish mosquito population grown. Such strategies like the one used in 2008 in Spain are disclosed here as a model. A multipronged approach using source reduction, larviciding treatments with *Bacillus thuringiensis israelensis* and diflubenzuron, adulticide treatments, and cleaning up uncontrolled landfills resulted in reduction of number of *Aedes albopictus* eggs in the areas with intervention. A substantial component for success was the community participation, which increased at each year of intervention [43].

Environmental management may be the most effective way to reduce mosquito-breeding sites and can be adapted to different settings. Studies in the US showcase the management of salt marshes used to create ponds and radial ditches as a way to reduce mosquito production and enhance fish predation on mosquitoes [44]. In other settings improving waste management, access to clean water, and providing better sewage services can also diminish breeding sites, however this approach must go along with urban planning.

#### Capacity building

Training of specialist and technicians in the use and implementation of tools for controlling the transmission of VBDs including modern technologies may result in improving risk assessment and disease control. One of these approaches is utilizing GIS mapping technology to track malaria transmission or *Aedes spp.* distribution. Such instruments allow for profiling of risk areas in accordance to different factors such as: temperature, land use and land changes, vector exposure, among others [45]. These technologies integrate environmental, agricultural, and health data and provide ways to plan treatment patterns accordingly to reduce mosquito breeding habitats and reducing risks of contracting a VBD [46,47]. Rapid diagnostic tools, specifically for malaria programs necessitates to be adapted to local environments and utilized by local workers as control strategies campaigns during severe epidemiological and entomological manifestations [48].

For effective application of control methods both programs proved that community involvement was key for their success; consequently, stakeholders should strengthen investments in community education. Studies done in neighborhoods of North Carolina revealed that 95% of the residents, who had not previously experienced any form of outreach from the local MCP, perceived that the door-to-door awareness evaluation was an effective form of outreach. This study also identified that many residents were unaware that mosquitoes could breed in their own backyards [49]. A low risk perception in the community underestimates the high danger potential of VBDs, which also may impact the effectiveness of public health interventions [50].

#### Advocacy, social mobilization, and legislation

The IVM analysis results further promotes knowledge sharing from social ecological standpoint. To promote the interaction of the community with decision makers and health leaders can lead to sustainable solutions and creating sustainable policies [51,52]. Knowledge exchange should lead to tangible outcomes starting from a bottom up approach creating policies for each community that are also enforced at the local level. Two country examples are worth reviewing. Singapore’s joint policies on source reduction for *Aedes aegypti* population control that include public education, and law enforcement has made the control of dengue much more manageable [53]. Thailand instituted infrastructural modifications that integrated dengue control into regular healthcare practices at the local level. Now dengue control is a priority in health plans of the local Thai government. Careful implementation of local code abatements, notification of breeding sites by citizens, addressing issues of waste management disposal, proper maintenance of public spaces can aid in the definition of legislation that once implemented and followed can improve overall environmental management and identify better application strategies for vector control [54].

#### Collaboration among sectors

Globally, the control and elimination of VBDs requires inter-sectorial and multidisciplinary policies that connect public health data with targeted interventions. To execute such plans knowledgeable political and health leaders should guide integrated approaches at different organizational levels i.e. integrate state with city law, further adapting lessons learned from data and monitoring into national health systems and the local communities [55]. To make a broad impact on the health and quality of life of populations at risk of VBDs, multiple stakeholders need to expand, maintain, and integrate MCPs to public health surveillance systems [56].

## Conclusion

Given that AMCD was created mainly out of the necessity to control nuisance-biting mosquitoes, the American MCP makes a strong emphasis on pest management. Special considerations on virus surveillance include that many dengue and WNV may be asymptomatic and therefore undiagnosed. Still, it is remarkable that no human cases from WNV have been reported in Saint Johns County since 2003, even though the virus circulates among non-human hosts. Alternatively, SNEM was developed based on the necessity to mitigate disease and protect the economy of residents; therefore, their emphasis is mainly in human disease and management. The epidemiological and entomological approach of both sites makes up for the main strengths of each program and provides the first finding of our analysis. Entomological and epidemiological surveillance systems are equally important components that need to be included in MCPs daily operations but they have the potential to be more efficient if both systems are integrated.

To recommend standard strategies is not realistic because MCPs activities differ in locations and circumstances; therefore the continuous analysis of the problems and constraints in MCPs is crucial to their own success [57]. Our SWOT analysis supports continuing local systematic evaluations of the MCPs and sharing the knowledge of successful and failed control strategies to ultimately provide dynamic solutions in different settings.

Nonetheless, our analysis was structured in an IVM framework, which can be applied in any setting and further guides political and health leaders to streamline MCPs activities into their own public health systems. The comparative analysis of MCPs in Saint Johns County, Florida and Guayas, Ecuador uses principals of IVM to develop recommendations for different health systems that may face similar challenges. Overall, the information gathered from residents and vector control experts at both institutions suggests that government administrations need to provide better environmental management, municipal infrastructure, technologies, and community participation to enhance MCPs activities and improve the quality of life of residents.

## Abbreviations

MCP: Mosquito control programs; VBD: Vector borne diseases; WHO: World Health Organization; IVM: Integrated vector management; AMCD: Anastasia Mosquito Control District; SNEM: *Servicio Nacional de Control de Enfermedades Transmitidas por Vectores Atrópodos*; SWOT: Strength, weaknesses, opportunities, and threats; USAID: United States Agency for International Development; WNV: West Nile virus; FDACs: Florida Department of Agriculture and Consumer Services; SIVE *alerta*: *Sistema Integrado de Vigilancia Epidemiológica*; PAHO: Pan American Health Organization; NGOs: Non-governmental organizations; INSPI: *Instituto Nacional de Salud Pública e Investigación*; SENESCYT: Secretaría Nacional de Educación Superior, Ciencia, Tecnología e Innovación; ENSO: El Nino Southern Oscillation; GIS: Geographical Information Systems.

## Competing interests

The authors declare that they have no competing interests.

## Authors’ contributions

DPN, WAQ, RDX, and JCB conceptualized, interpreted, and wrote the first draft of this research. Data collection and project execution was possible through HJ, JCP, and EG. All authors read and approved the final manuscript.

## Authors’ information

DPN is a PhD student in the Department of Public Health Sciences. She conducted research studies at the Anastasia Mosquito Control District (AMCD) and the *Servicio Nacional de Control de Enfermedades Transmitidas por Vectores Atrópodos* (SNEM) as part of her dissertation.

WAQ is a Post-Doctoral Fellow at the University of Miami in Miami, FL. WAQ earned her Ph.D. in Medical Entomology from University of Florida and a Masters degree in Entomology from Auburn University. Her work started up at the Anastasia Mosquito Control District, but her experience has expanded internationally through China, the Middle East, Africa and Latin America. WAQ has over 30 publications and book chapters related to vector control and pathogen transmission.

HJ is a Doctor in Medicine and one of the main operational directors SNEM. He has over 25 years worth of experience in the vector control program of Ecuador and currently teaches as a Infectious Diseases professor at *Universidad Estatal de Guayaquil, Facultad de Medicina*.

JCP, Executive Director of the Ecuadorian *Instituto Nacional de Salud Pública e Investigación* (INSPI) in 2012, is both a Medical Doctor and a Doctor in Law graduated from *Universidad Católica Santiago de Guayaquil*. Additionally, he earned two Masters degrees in Hospital Administration and Behavioral and Biological Neuroscience.

RDX is the Director of the AMCD. He earned a Medical Doctor and a Ph.D. in Entomology degrees in China. RDX work focuses on applied research and control operations of mosquito control programs. The scope of his experience deals with administration of programs, development of research, and training of administrators, field workers, and students.

EG is a Professor in Tropical Medicine and Infectious Diseases at the *Universidad Católica Santiago de Guayaquil*. He is the lead representative for dengue at SNEM. He investigates community based participatory research for dengue control.

JCB is a Senior Professor and Chief of the Division of Environment & Public Health in the Department of Public Health Sciences at the University of Miami. His research focuses on the ecology and control of multiple vector-borne diseases, including malaria and dengue. He is a leader in the research of vector borne diseases and interacts with international collaborators and University of Miami staff, faculty, and students from several departments and centers. Dr. Beier’s work is interdisciplinary and geared towards disease-endemic countries. He has over 250 publications and is the editor of Acta Tropica.

## Pre-publication history

The pre-publication history for this paper can be accessed here:

http://www.biomedcentral.com/1471-2458/14/674/prepub
